# Asparagus Decline and Replant Problem: Autotoxicity, Autotoxic Substances, and Their Biological Functions

**DOI:** 10.3390/biology15070537

**Published:** 2026-03-27

**Authors:** Hisashi Kato-Noguchi, Midori Kato

**Affiliations:** Department of Applied Biological Science, Faculty of Agriculture, Kagawa University, Miki, Kagawa 761-0795, Japan

**Keywords:** allelopathy, cinnamic acid, decomposition, defense mechanism, *Fusarium*, mycotoxin, phenylpropanoid, phytotoxicity, root residue

## Abstract

The cultivation of asparagus (*Asparagus officinalis* L.) is plagued by two serious issues: “asparagus decline” and “asparagus replant problem”. The average lifespan of an asparagus plant is 15 to 20 years. However, its productivity decreases after a few years (asparagus decline). Even when these asparagus plants are replaced with new ones, the new plants remain unproductive (asparagus replant problem). The main causes of these problems are a *Fusarium* infection and asparagus autotoxicity. Several reviews have been conducted on *Fusarium*. Despite the accumulation of evidence on asparagus autotoxicity in the literature over the past four decades, no review has focused specifically on asparagus autotoxicity. It has been reported that asparagus growth is inhibited by asparagus root residues, leachates, root exudates, and rhizosphere soils. Several phenylpropanoids, including *trans*-cinnamic acid, *p*-coumaric acid, caffeic acid, and ferulic acid, have been identified as asparagus autotoxic substances in these root residues, root exudates, rhizosphere soils, growth media, and/or plant tissues. Tryptophan, 3,4-methylenedioxycinnamic acid, and iso-agatharesinol were also identified as asparagus autotoxic substances. These substances may cause autotoxicity by disrupting phytohormone levels, cellular metabolism, impairing membrane function, and by inducing oxidative stress. Although cinnamic, *p*-coumaric, caffeic, and ferulic acids have been reported to act as antibiotics, these compounds have also been shown to weaken the defense mechanisms of asparagus against pathogen infection, and enhance the *Fusarium* pathogenicity. The presence of these autotoxic substances, coupled with a *Fusarium* infection, may create a vicious cycle that worsens “asparagus decline” and “asparagus replant problem”. This is the first review to focus on the asparagus autotoxicity.

## Simple Summary

The cultivation of asparagus (*Asparagus officinalis* L.) is plagued by two serious issues: “asparagus decline” and “asparagus replant problem”. These issues are characterized by the low vigor, growth, and productivity. It is believed that these issues result from a combination of a *Fusarium* infection and asparagus autotoxicity. Over the past four decades, evidence of asparagus autotoxicity has been published. However, no review has focused specifically on this autotoxicity. This paper provides an overview of the literature on asparagus autotoxicity, including the substances and mechanisms involved. The growth of asparagus has been reported to be inhibited by asparagus root residues, leachates, root exudates, and rhizosphere soils. Several phenylpropanoids, including *trans*-cinnamic acid, *p*-coumaric acid, caffeic acid, and ferulic acid, have been identified as autotoxic substances in asparagus root residues, root exudates, rhizosphere soils, growth media, and/or plant tissues. Tryptophan, 3,4-methylenedioxycinnamic acid, and iso-agatharesinol were also identified as its autotoxic substances. These substances may cause autotoxicity by disrupting phytohormone levels, cellular metabolism, membrane function, and by inducing oxidative stress. These substances also weaken the defense mechanisms of asparagus against pathogen infection, and enhance *Fusarium* pathogenicity. Therefore, these substances may be responsible for asparagus autotoxicity.

## 1. Introduction

**Figure 1:**
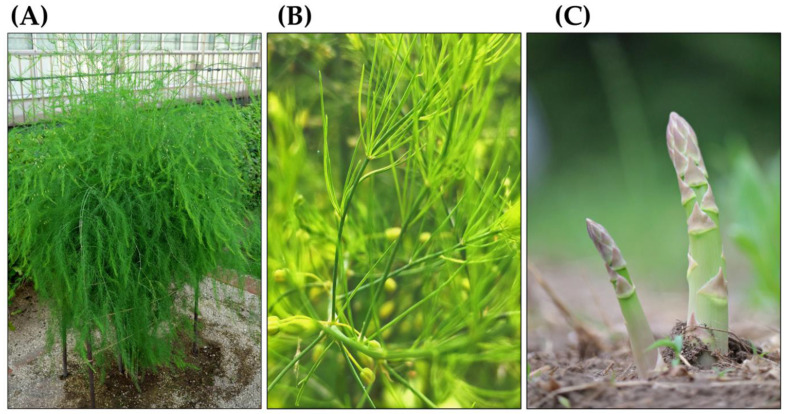
*Asparagus officinalis*. (**A**) Above grand part (Fern), (**B**) Branches and cladodes (Leaves), (**C**) Spears (Young shoots).

**Figure 2:**
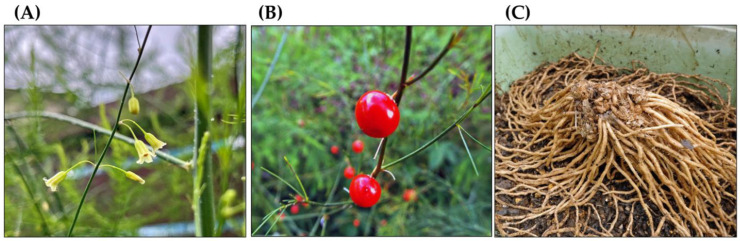
*Asparagus officinalis*. (**A**) Flowers, (**B**) Fruits, (**C**) Roots and rhizomes.

Asparagus (*Asparagus officinalis* L.), formerly classified in the Liliaceae family, is a perennial herb that now belongs to the Asparagaceae family [1]. It grows to a height of 1–2 m and has glabrous and erect stems that branch well and bear feathery foliage called a fern. Its leaves have degenerated into needle-like cladodes. These cladodes are 5–40 mm long, 1 mm wide, and clustered in whorls of 2–15 cladodes. Bell-shaped flowers are produced at the bases of the cladodes. These yellowish-green flowers are approximately 5 mm long. They consist of six petals. This species is dioecious. Male flowers have six stamens, while female flowers have a pistil and vestigial stamens. The fruit is 6–9 mm in diameter, turns red when ripe, and contains 2–4 seeds. Its underground parts consist of rhizomes and roots, known as crowns. Crowns are usually planted instead of seeds to grow new plants for cultivation. Green young shoots, known as spears, arise from the rhizomes and are harvested as a vegetable. White asparagus is not a variety of *A. officinalis.* It is produced by keeping the spears in the dark [1,2,3,4,5,6,7] (Figure 1 and Figure 2).

Asparagus is native to the Mediterranean region and Western Asia [1,2]. It has been harvested for thousands of years by several ancient civilizations, including the Egyptians, Greeks, and Romans, for use as both a vegetable and a medicine [8]. It is currently widely cultivated as a vegetable crop in well-drained soils in temperate and subtropical climates. Its global production has steadily increased due to the growing demand for nutritious vegetables. It also contains various functional ingredients, including dietary fibers, polyphenols, saponins, and anthocyanins [9,10,11,12]. Global production of asparagus reached 8.6 million tons in 2023. China accounted for 88% of the total production, followed by Peru (5.5%), Mexico (3.9%), Germany (1.3%), and Spain (0.5%) [13,14,15].

The average lifespan of an asparagus plant is 15 to 20 years, and its spears can be harvested for over 10 of those years. However, a major problem in asparagus cultivation is the gradual decrease in productivity known as the “asparagus decline”, as defined by Grogan and Kimble [16]. Asparagus reaches its peak production several years after cultivation begins, and then gradually decreases year by year. Typical symptoms include smaller spears and fewer sprouts. The above-ground parts, including the stems, branches, and cladodes, become chlorotic and stunted. The roots often shrivel or disappear [17,18,19,20]. Even when old asparagus plants in a field are replaced with new asparagus plants, the vigor, spear quality, and productivity of the replaced new asparagus plants remain relatively low compared to plants in areas where asparagus has never been cultivated. This phenomenon is known as the “asparagus replant problem” [17,21,22,23].

The “asparagus decline” and “asparagus replant problem” are thought to be caused by a combination of biotic and abiotic stress factors. Biotic factors include pathogenic fungi and viruses. The primary biotic factor is *Fusarium* infection [17,18,19,20,23,24,25]. There are at least 300 species of *Fusarium* taxa [26], but the most commonly isolated species from asparagus is *Fusarium oxysporum* f. sp. *asparagi* (thereafter *Fusarium oxysporum*), followed by *Fusarium proliferatum*. These *Fusarium* infestations cause diseases such as rot, wilt, and blight in asparagus plants, including its seedlings [22,27,28,29,30,31]. These diseases reduce the vigor, spear quality, productivity, and lifespan of asparagus plants [32,33,34]. These *Fusarium* species produce spores in humid conditions. These spores are then distributed throughout entire asparagus fields. *Fusarium oxysporum* can survive in soil containing asparagus residue for over 25 years, even in the absence of asparagus plants [35,36]. These *Fusarium* species also form chlamydospores, which are thick-walled spores that survive much longer than regular spores [37,38]. The asparagus miner (*Ophiomyia simplex,* Agromyzidae family) is a stem-mining fly that specializes in asparagus and is widely distributed. It acts as a vector for *Fusarium* infection in asparagus and is responsible for the development of *Fusarium* diseases [39,40].

The *Fusarium* infection process begins when spores and chlamydospores germinate in the soil and form hyphae [41]. These hyphae adhere to the root surfaces of their hosts. The hyphae recognize their hosts through compounds in the plant cutin layers, such as dihydroxy-C16 and trihydroxy-C18 acids. These compounds, which are unique to suitable hosts, stimulate *Fusarium* to produce cell wall-degrading enzymes, including cutinase, pectinase, cellulase, lipase, and xylanase. These induced enzymes break down the plant cell wall, enabling *Fusarium* to enter the root epidermis. The fungus then spreads into the cortex and xylem of the host plant roots. From there, it spreads further to the storage roots, crowns, and stems [36]. These cell wall-degrading enzymes are also involved in the pathogenesis. The infected fungi destroy plant tissue and absorb nutrients from their host plants [42,43,44,45,46,47].

*Fusarium* species secrete several types of effector proteins, into host plant cells, including SIX effector proteins, small secreted effector proteins (SSP), and FOLD effectors. Effector proteins are cysteine-rich proteins consisting of 50 to 300 amino acid residues [46,48,49]. Effector proteins act as molecular probes that inhibit the production of host plant proteins involved in defense mechanisms against pathogen infection. They also alter the expression of host plant genes. These altered genes enable *Fusarium* to plunder nutrients from host plants and prevent the apoptosis in host plant cells during infection [50,51]. Therefore, effector proteins interfere with the defense mechanisms and alter metabolism of host plants, thereby facilitating *Fusarium* pathogenicity.

Pathogenic *Fusarium* species also produce several mycotoxins, including moniliformin, fumonisins, enniatins, and beauvericin. The production of these mycotoxins depends on the intrinsic and extrinsic conditions [52,53,54,55]. Moniliformin, enniatins A1 and B, beauvericin, and fumonisins B_1_, B_2_, and B_3_ were found in asparagus infected by *F. oxysporum* and *F. proliferatum* [31,47,56,57,58]. Moniliformin has been shown to suppress plant growth by reducing photosynthetic pigments [59]. It also suppresses leaf development and seedling growth [60]. The toxicity of enniatins and beauvericin is related to their ionophoric properties, which increase cellular membrane permeability to ions. These compounds induce oxidative stress. They also cause DNA fragmentation and chromosomal aberrations, resulting in apoptosis [61,62,63]. Beauvericin reduces ascorbic acid metabolism and disrupts the ascorbate system, ultimately resulting in cell death [64]. Over 15 different structures of fumonisins have been identified and classified into groups A, B, C and P. Fumonisin B_1_ is the most abundant [65,66]. It blocks the biosynthesis of sphingolipids by inhibiting ceramide synthase, which is a key enzyme in lipid metabolism. Sphingolipids play important roles in signal transduction and cell recognition. Therefore, fumonisin B_1_ interferes with these functions by inhibiting sphingolipid biosynthesis [67,68,69]. Fumonisin B_1_ induces toxicity by causing oxidative stress, apoptosis, and alterations in cytokine expression [70]. Fumonisins degrade the endosperm and reduce the protein matrix surrounding the starch granules [71]. They also significantly inhibit the growth of maize roots [72]. These mycotoxins often contaminate grains of crops, such as maize, wheat, and barley, that have been infected by *Fusarium.* Mycotoxins can also cause disease and death in animals and humans [61,73,74].

**Figure 3:**
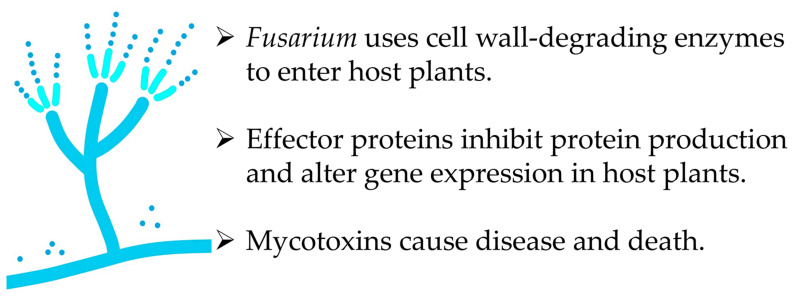
*Fusarium* infection and toxicity.

As described above, *Fusarium* alters the metabolism and defense mechanisms of host plants against pathogen infection by producing effector proteins and mycotoxins. It can also destroy their plant tissue by producing several cell wall-degrading enzymes. These enzymes, effector proteins, and mycotoxins enhance *Fusarium* pathogenicity, resulting in the reduced asparagus quality and production (Figure 3). Several reviews have summarized the effects of a *Fusarium* infection as a biotic factor that suppresses asparagus production [36,39,47,75,76].

Abiotic stress factors that contribute to the “asparagus decline” and “asparagus replant problem” include autotoxicity, climate, water availability, overharvesting, and soil conditions such as nutrient and pH levels, and soil compaction. Of these factors, autotoxicity of asparagus is considered the primary abiotic stress factor [17,19,77]. Other factors, such as climate, water availability, overharvesting, and soil conditions affect not only asparagus, but also the growth and productivity of many other plants.

Over time, autotoxic substances accumulate in the soil of the asparagus rhizosphere. Excessive accumulation of these substances suppresses the asparagus growth and vigor, resulting in the “asparagus decline” and “asparagus replant problem”. Over the past four decades, evidence of asparagus autotoxicity has been published. However, no review has specifically focused on this phenomenon. This paper provides an overview of the literature on asparagus autotoxicity, including the substances and mechanisms involved, and the areas in which more information is needed. A combination of online search engines was used to search the literature: Scopus, PubMed, Springer Link, ScienceDirect, and Google Scholar. The following terms relating to asparagus were searched: autotoxicity, allelopathy, *Fusarium*, mycotoxin, secondary metabolite, residues, root exudate, growth inhibition, and agriculture. We included these research papers in our analysis as thoroughly as possible. However, we excluded publications with unclear methods or no statistical analysis. It begins by discussing autotoxicity and its ecological significance.

## 2. Autotoxicity and Its Ecological Significance

Autotoxicity is an example of intraspecific allelopathy. Allelopathy is the chemical interaction between a donor species and another species. The donor species produces allelochemicals, that are released into the surrounding environment, including the soil of the rhizosphere. These allelochemicals are released through volatilization from the plants and leaching from their aerial parts by rainfall. They are also released through root exudation and the decomposition process of plant residues in the soil [78,79,80,81,82,83]. Allelopathy has also been demonstrated in fern and moss species [84,85,86]. These allelochemicals can suppress the germination, growth, physiological processes, and reproduction of other plant species. Autotoxic substances, or autotoxins, act like allelochemicals. However, unlike allelochemicals, they suppress the germination, growth, physiological processes, and reproduction of the same species of donor plants, as well as the donor plants themselves [78,87,88].

Allelopathy gives donor plants a competitive advantage over neighboring plants. Plants compete with neighboring plants for light, nutrients, and water. Allelopathy provides an advantage in this resource competition by suppressing the germination and growth of other plant species [78,79,89]. Some invasive plant species, such as *Pueraria montana* var. *lobata*, *Acacia mearnsii*, *Sphagneticola trilobata*, and *Ulex europaeus*, which are listed among the world’s 100 worst invasive alien species [90], exhibit significant allelopathic activity. This allelopathy contributes to their invasive characteristics [91,92,93,94].

Autotoxicity has been observed in many crop plants, including alfalfa (*Medicago sativa* L.), sugarcane (*Saccharum officinarum* L.), and mung bean (*Vigna radiata* (L.) Wilczek). Several autotoxic substances, including *p*-coumaric acid, *p*-hydroxybenzoic acid, salicylic acid, ferulic acid, vanillic acid, and/or syringic acid have been identified in these herbaceous plants [78,87,95,96,97,98]. Declines in production and replanting issues have been observed during the cultivation of apple (*Malus pumila* Mill.), peach (*Prunus persica* (L.) Batsch), citrus (*Citrus* spp.), and kiwifruit (*Actinidia deliciosa* (A.Chev.) A.Chev.). Amygdalin, abscisic acid-β-D-glucopyranoside, phlorizin, phloretin, epicatechin, *p*-coumaric acid, and/or benzoic acid have been identified as autotoxic substances in these woody plants [99,100,101,102,103]. Most of these autotoxic substances have also been identified as allelochemicals in many other plant species [78,79,80,103]. This has provided insight into their role in plant biology. Unlike allelopathy, however, autotoxicity acts as a form of self-regulation. It controls population density to prevent intense competition for resources within the same species, and suppresses germination during adverse environmental conditions. It influences the migration of species from one habitat to another. It also increases species diversity by reducing the population density of host plant species within specific habitats [78,87,88,104]. However, more information is needed to understand the ecological significance of plant autotoxicity.

## 3. Asparagus Autotoxicity

Similar to allelochemicals, autotoxic substances are released into the surrounding environment through volatilization, leaching, root exudation and the decomposition process of plant residues [78,82,85,99,100,101]. Asparagus autotoxicity has been identified in plant residues, leachate from the plants, root exudates, and soil in which asparagus has previously grown.

### 3.1. Autotoxicity of Asparagus Residues

The biomass of asparagus root residues in the soil was 4180–11,060 and 420–1140 kg of dry weight per hectare after one year and 10 years, respectively, following the termination of asparagus cultivation [21]. Therefore, a significant amount of residue remained in the soil even after 10 years. Soil mixed with fumigated asparagus roots suppressed the germination of asparagus seeds [105]. In a laboratory setting, sterilized asparagus root fragments also exhibited an inhibitory effect [21]. Under greenhouse conditions, root amendments of asparagus suppressed the growth and its nutrient uptake of asparagus, including its uptake of phosphorus, nitrogen, potassium, calcium, and magnesium [106]. These inhibitory effects were caused by the sterilized asparagus residues. Therefore, the inhibitory effects of root residues are caused by more than just the presence of pathogen in the residues. One possible cause of the inhibition may be the presence of compounds in the residues. These compounds may be released into the soil during the decomposition process of residues, and suppress the growth and nutrient uptake of asparagus. These findings suggest that the “asparagus decline” may also be caused by the accumulation of root residues in asparagus fields.

Three-month-old asparagus plants were transplanted into the soil mixed with sterilized asparagus roots and crowns. This treatment inhibited the growth of the transplanted asparagus. The inhibitory effect of root residues was greater than that of crown residues [107,108]. Asparagus seedlings were transplanted into a sandy soil mixture containing asparagus root and stem residues. This resulted in inhibition of growth of the transplanted seedlings. The inhibitory effect of the root residues was greater than that of the shoot residues [109]. These investigations suggest that asparagus root, crown, and stem residues can suppress the growth of transplanted asparagus. Root residues were more effective than shoot and crown residues. Therefore, these residues may also contribute to the “asparagus replant problem”. The inhibitory effects of root residues are greater than those of shoot and crown residues.

### 3.2. Autotoxicity of Asparagus Leachates and Root Exudates

After applying the nutrient solution, the resulting leachates were collected from the asparagus plants. Then, these leachates were applied to another batch of asparagus plants. This resulted in growth suppression of the asparagus plants [110]. The nutrient solution previously used for hydroponic asparagus cultivation hindered the growth of the roots and shoots of new asparagus seedlings. These experimental conditions rule out nutrient deficiency as the cause [111]. Root exudates were collected from one-year-old asparagus plants grown in a vermiculite medium, and applied to other one-year-old asparagus plants. This treatment suppressed the root and shoot growth of asparagus seedlings under greenhouse conditions [112]. These findings suggest that asparagus may release autotoxic substances into the soil through root exudation and leaching.

Additionally, the first group of asparagus seeds was germinated and grown in an agar medium for 56 days. After harvesting all of the seedlings from the first group, the second group of asparagus seeds was sown on the same medium and grown for 56 days. This treatment suppressed the fresh and dry mass growth of the second group of seedlings. The nutrient uptake of the second group of seedlings, including phosphorus, nitrogen, potassium, calcium, and magnesium, was also suppressed [113]. This observation suggests that the first group of asparagus seedlings releases autotoxic substances, which accumulate in the growth medium. These substances then inhibit the growth of the second group of the seedlings by suppressing their nutrient uptake.

### 3.3. Autotoxicity of Asparagus Rhizosphere Soil

Rhizosphere soils, in which asparagus had been cultivated for over 10 years, inhibited the root and shoot growth of asparagus seedlings. However, the pH, electrical conductivity, and concentrations of nitrogen, phosphorus, potassium, magnesium, and calcium in these soils were not significantly different from those in soils that did not exhibit inhibitory effects on growth [114]. Rhizosphere soils were collected from asparagus fields, sieved to remove plant residues, and then extracted using an aqueous methanol solution. These extracts inhibited the root and shoot growth of asparagus seedlings [23]. Acetone and methanol extracts of asparagus soil inhibited root and shoot growth of asparagus seedlings in a concentration dependent manner. The inhibitory effects of the acetone and methanol extracts were similar [109]. These findings suggest that asparagus rhizosphere soils may contain autotoxic substances, and these compounds are exactable.

## 4. Autotoxic Substances in Asparagus Plants and Residues

As discussed in Section 3, asparagus root residues, leachates, root exudates, and rhizosphere soils exhibited autotoxicity, which suggests the presence of autotoxic substances. These substances can be extracted from plant tissues and residues because they are produced and stored in the plant tissues until they are released [78,82,85,99,100,101].

Aqueous extracts obtained from the stems, crowns and roots of field-grown and tissue-cultured asparagus plants inhibited the germination and growth of asparagus. The field-grown asparagus were collected from 4–5 year-old asparagus fields. The tissue-cultured asparagus were grown on a Murashige and Skoog medium for two years and remained uninfected by pathogens during incubation. The inhibitory effects were greater in the extracts from the tissue-cultured asparagus than in the extracts from the field-grown asparagus. Both root extracts were more effective than other extracts [115]. These results suggest that the inhibitory effects are not caused by pathogen contamination in the extracts. Asparagus plants may contain autotoxic substances in their stems, crowns and roots. The roots likely contain more of these autotoxic substances than the stems and crowns.

Aqueous extracts from root residues have been shown to inhibit the growth of asparagus seedlings [21]. Aqueous extracts and aqueous methanol extracts of asparagus roots and shoots also inhibited the growth of asparagus seedling in a concentration dependent manner [23,109]. Aqueous extracts of asparagus roots increased membrane electrolyte efflux and inhibited the peroxidase activity of asparagus seedlings [108]. These findings suggest that fresh asparagus roots and shoots, and residues, may contain autotoxic substances, and some of them increase electrolyte efflux and inhibit the peroxidase activity. These substances can be extractable with water and aqueous methanol.

Asparagusic acid and its related compounds have been isolated from asparagus shoots [116,117]. These compounds have demonstrated fungicidal, antibacterial, nematocidal, insecticidal, and herbicidal activity [118]. However, the role of asparagusic acid and related compounds in asparagus autotoxicity remains unclear.

**Figure 4:**
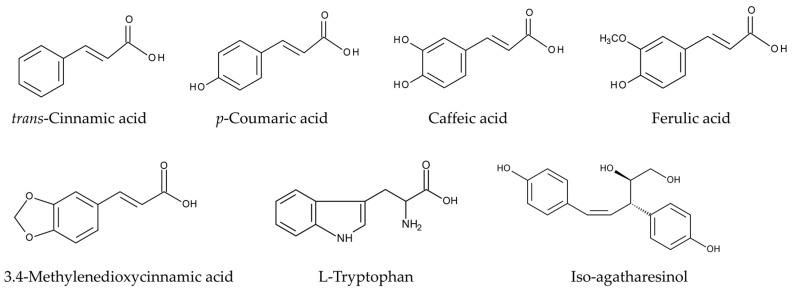
Autotoxic substances identified in *Asparagus officinalis*.

Phytotoxic substances, including ferulic acid, isoferulic acid, malic acid, fumaric acid, and 3,4-methylenedioxycinnamic acid, were isolated from the aqueous extracts of asparagus roots. Although the autotoxic activity of these compounds on asparagus was not determined, 3,4-methylenedioxycinnamic acid was the most active against the seedling growth of cress (*Lepidium sativum* L.). It inhibited over 50% of cress roots and shoots at a concentration of 50 ppm (260 μM) [119]. 3,4-Methylenedioxycinnamic acid is a derivative of cinnamic acid. It effectively inhibits 4-coumarate-CoA ligase (4CL) in the phenylpropanoid pathway. This pathway is a vital metabolic process in plants [120,121,122]. The growth inhibitory activity of ferulic acid on asparagus was reported later. Exogenously applied ferulic acid was found to suppress the growth of asparagus plants [123]. Its action mechanisms have also been reported as follows: Ferulic acid increases cell membrane permeability by stimulating lipid peroxidation, which leads to electrolyte leakage. It also suppresses photosynthesis by reducing chlorophyll content. Ferulic acid esterifies cell wall polysaccharides and interferes with lignin synthesis, making the cell wall inflexible. This physically restricts cell expansion [124,125]. Ferulic acid also suppressed the spore germination and growth of the arbuscular mycorrhizal fungus *Glomus fasciculatum* in vitro. It also inhibited the colonization of *G. fasciculatum* in asparagus roots [126]. Arbuscular mycorrhizal fungi contribute to many aspects of plant growth and development, particularly by improving nutritional conditions, increasing stress tolerance, and enhancing disease resistance [127,128]. Consequently, inhibition of colonization of *G. fasciculatum* causes the growth suppression of host plants. Therefore, ferulic acid may suppress the growth of asparagus by disrupting cell membrane function, lignin synthesis, and photosynthesis. It may also inhibit the colonization of *G. fasciculatum*. Ferulic acid and 3,4-methylenedioxycinnamic acid may act as autotoxic substances in asparagus (Figure 4).

Aqueous root extracts of asparagus plants inhibited the roots and shoot growth of asparagus seedlings. Caffeic acid and tryptophan were identified as the active components in the extracts. The concentrations of caffeic acid and tryptophan were 160 and 180 mg/kg of fresh root weight, respectively. The inhibitory effect of tryptophan on the root and shoot growth of asparagus was greater than that of caffeic acid [23]. The inhibitory effects of tryptophan have also been reported in several other plant species [129,130]. Tryptophan is a precursor to the phytohormone indole acetic acid (IAA; auxin). Therefore, adding exogenous tryptophan may interrupt auxin biosynthesis [129,131]. However, the mechanism by which tryptophan inhibits growth remains unclear.

Caffeic acid has also been isolated from methanol extracts of asparagus roots. In laboratory experiments, it suppressed the germination of asparagus seeds both under sterile conditions and under conditions with *Fusarium* inoculation. It also suppressed asparagus germination in pots filled with pre-moistened peat-lite mix under greenhouse conditions [132]. The concentration of caffeic acid was determined to be 1.7–3.0 mg/g in dry roots [133], and 0.26–3.2 mg/g in dry asparagus rhizomes [134]. Caffeic acid induces an oxidative stress condition [135]. It also interrupts gibberellic acid biosynthesis and downregulates the MAPK signaling pathway [136]. Gibberellic acid is a phytohormone and involved in many essential physiological processes in plants [137,138]. The MAPK signaling pathway is involved in recognizing and responding to stress factors, including adverse environmental conditions, pathogen infection, and herbivore attacks [139,140]. Therefore, caffeic acid may act as an autotoxic substance, causing oxidative stress, and inhibiting the synthesis of gibberellic acid and the MAPK signaling pathway.

Aqueous methanol extracts of asparagus rhizomes inhibited the root and shoot growth of asparagus seedlings in a concentration dependent manner. The extract obtained from 7.4 and 21.5 mg of dry asparagus rhizomes produced 50% inhibition of root and shoot growth of asparagus seedlings, respectively [141]. The extracts were purified using a bioassay-guided purification process that monitored the autotoxic activity of each separated fraction at every step of the purification process. The most active fraction was then used for the next separation. This process resulted in the isolation of two autotoxic substances: *p*-coumaric acid and iso-agatharesinol (Figure 4). *p*-Coumaric acid and iso-agatharesinol inhibited the root and shoot growth of asparagus seedlings in a concentration dependent manner at concentrations greater than 0.1 mM. The IC_50_ values, indicating 50% growth inhibition, for *p*-coumaric acid were 0.36 and 0.53 mM for the roots and shoots of asparagus seedlings, respectively. The IC_50_ values for iso-agatharesinol were 0.62 and 0.72 mM for the roots and shoots of asparagus seedlings, respectively. *p*-Coumaric acid causes cellular membrane damage, inhibits photosynthesis, and induces oxidative stress conditions [135,142]. *p*-Coumaric acid has also been reported to be involved in the autotoxicity of tobacco (*Nicotiana* spp.) [143]. Iso-agatharesinol, a nor-lignan, is synthesized via the phenylpropanoid pathway [144]. It has also been isolated from *Asparagus cochinchinensis* [145], and exhibits allelopathic activity against several other plant species [141]. However, the mechanism of action of iso-agatharesinol on growth inhibition remains unclear.

Several flavonoids, including quercetin, rutin, and kaempferol, and saponins, have been identified in asparagus spears [146,147,148]. These studies examined the nutritional value and Functional ingredients of asparagus as a food source. Some flavonoids have also been reported to exhibit allelopathic activity [149]. However, growth inhibitory activity of these flavonoids on asparagus has not yet been determined. Therefore, their role in asparagus autotoxicity remains unclear.

As discussed in this section, *p*-coumaric acid, caffeic acid, ferulic acid, tryptophan, and iso-agatharesinol inhibited the germination and/or growth of asparagus. Ferulic acid also suppressed arbuscular mycorrhizal colonization, and 3,4-methylenedioxycinnamic acid inhibited the phenylpropanoid pathway. Therefore, these compounds may be involved in asparagus autotoxicity (Figure 4). However, the concentrations of these compounds in asparagus root exudates, residues, and rhizosphere soils remain unknown. They only act as autotoxic substances after being released into the environment from plants and residues. Further research on this process in these compounds is necessary to better understand asparagus autotoxicity.

## 5. An Autotoxic Substance in Asparagus Rhizosphere Soil and Root Exudation

**Figure 5:**
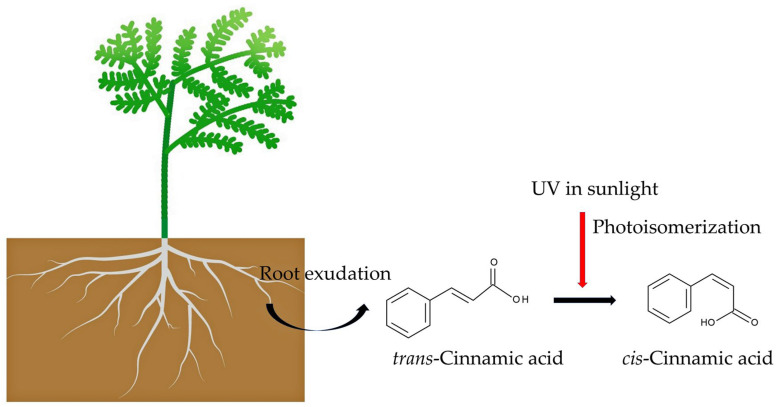
Asparagus root exudation and photoisomerization. Asparagus exudes *trans*-cinnamic acid into soil. *trans*-Cinnamic is photoisomerized by UV light in sunlight into *cis*-cinnamic acid. This isomerization increases the growth inhibitory activity by over 10-fold.

The soil was obtained from the rhizosphere of 10-year-old asparagus plants that were cultivated in a greenhouse. Aqueous methanol extracts of these rhizosphere soils inhibited the root and shoot growth of asparagus seedlings. The extracts obtained from 148 and 218 mg of soil produced 50% growth inhibition of the asparagus roots and shoots, respectively [150]. These extracts were purified using a bioassay-guided process. The purification process resulted in the isolation of *trans*-cinnamic acid (Figure 5), which inhibited the root and shoot growth of asparagus seedlings at concentrations greater than 10 μM. The IC_50_ values for *trans*-cinnamic acid were 24.1 and 41.6 μM for asparagus roots and shoots, respectively. The concentration of *trans*-cinnamic acid in the rhizosphere soil of 10-year-old asparagus plants was 7.5 mg/kg of soil [150]. This is equivalent to 51 μmol/kg of soil, considering the molecular mass of *trans*-cinnamic acid is 148.161 g/mol. The moisture content of the soil obtained from the rhizosphere of 10-year-old asparagus plants was 29.7% (*v*/*w*), equivalent to 297 mL of water per kg of soil [150]. Therefore, the concentration of *trans*-cinnamic acid in soil water is estimated to be 172 μM. This concentration of *trans*-cinnamic acid in the soil water exceeds the IC_50_ value required for 50% growth inhibition. Therefore, the accumulation of *trans*-cinnamic acid in the rhizosphere soil of 10-year-old asparagus plants can cause the growth inhibition of asparagus.

The asparagus seeds were germinated and grown on a Murashige and Skoog medium. On day 20, *trans*-cinnamic acid was found in the medium at a rate of 36 μg per plant [150]. This finding suggests that the asparagus releases *trans*-cinnamic acid into the medium. Plants can release a wide range of compounds from their root cells into the rhizosphere through the mechanisms involving plasmalemma-derived, endoplasmic-derived, and proton-pumping exudation [79]. Additionally, 10-year-old asparagus plants contained 5.7 and 4.2 g of *trans*-cinnamic acid per kg of roots and shoots, respectively [150], which suggests that asparagus produces and stores *trans*-cinnamic acid in its tissues. Therefore, asparagus plants may produce *trans*-cinnamic acid and release it into the growth medium, as well as rhizosphere soil, where it accumulates. *trans*-Cinnamic acid may also be released into the soil from asparagus residues during decomposition because it is present in the asparagus roots and shoots.

*trans*-Cinnamic acid increases the disruption of phytohormone indole-3-acetic acid (IAA) levels in plants due to increased IAA oxidase activity [151,152]. It inhibits plasma membrane H^+^-ATPases, which results in the suppression of proton transport and nutrient uptake [153,154]. *trans*-Cinnamic acid also induced oxidative stress conditions by increasing levels of reactive oxygen species and caused cellular damage [155,156].

Cinnamic acid primarily exists in plants as the stable form of *trans*-cinnamic acid. However, UV and sunlight irradiation cause it to isomerize into *cis*-cinnamic acid [157,158]. *cis*-Cinnamic acid exhibited over 10 times the growth inhibitory activity of *trans*-cinnamic acid [157]. *cis*-Cinnamic acid suppresses IAA biosynthesis and disturbs IAA transport in plants, causing growth inhibition [158]. *trans*-Cinnamic acid in soil can be converted to *cis*-cinnamic acid through isomerization when it is exposed to UV light from sunlight during certain cultivation practices. This process enhances the autotoxicity of asparagus by increasing the inhibitory activity of *trans*-cinnamic acid (Figure 5).

**Figure 6:**
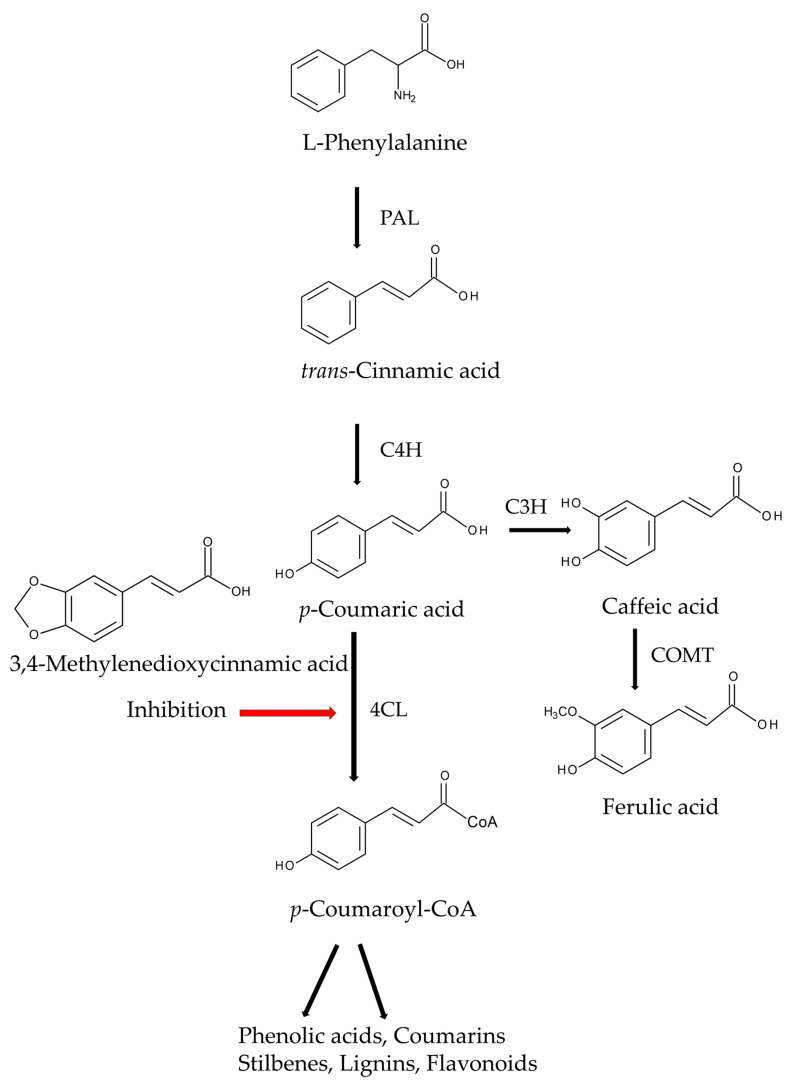
Metabolic pathway of phenylpropanoids in plants. PAL; Phenylalanine ammonia-lyase, C4H; Cinnamate 4-hydroxylase, 4CL; 4-Coumarate-CoA ligase, C3H; *p*-Coumarate 3-hydroxylase, COMT; Caffeic acid O-methyltransferase.

*trans*-Cinnamic acid is the first intermediate in the phenylpropanoid pathway. The pathway primarily begins in the cytoplasm, with the conversion of the amino acid L-phenylalanine into *trans*-cinnamic acid by phenylalanine ammonia-lyase (PAL). *trans*-Cinnamic acid is converted into *p*-coumaric acid by cinnamate 4-hydroxylase (C4H). *p*-Coumaric acid is then converted into *p*-coumaroyl-CoA by 4-coumarate-CoA ligase (4CL). *p*-Coumaroyl-CoA is a key precursor in the biosynthesis of essential plant compounds, such as phenolic acids, coumarins, stilbenes, lignins, flavonoids, and other phenylpropanoids. Caffeic acid (3,4-dihydroxycinnamic acid) is synthesized from *p*-coumaric acid by *p*-coumarate 3-hydroxylase (C3H). It is then converted into ferulic acid by caffeic acid O-methyltransferase (COMT) [159,160,161]. Therefore, these phenylpropanoids and/or their biosynthesis pathway may play an important role in asparagus autotoxicity (Figure 6).

3,4-Methylenedioxycinnamic acid is an effective inhibitor of 4-coumarate-CoA ligase [120,121,122]. It was identified in asparagus roots [119]. However, it is unlikely that endogenous 3,4-methylenedioxycinnamic acid interferes with the phenylpropanoid pathway in asparagus. This is because plants separate toxic substances from metabolism in the cytoplasmic matrix and store them in vacuoles [162,163]. To understand its role in asparagus autotoxicity, the presence of 3,4-methylenedioxycinnamic acid in asparagus rhizosphere soils should be determined.

As described in this section, *trans*-cinnamic acid was identified in asparagus plants and in the soil of their rhizosphere. It was also released form asparagus into their growth medium. *trans*-Cinnamic acid inhibited the growth of asparagus roots and shoots. Therefore, *trans*-cinnamic acid may be involved in asparagus autotoxicity. Additionally, sunlight causes the isomerization of *trans*-cinnamic acid into *cis*-cinnamic acid, which increases its inhibitory activity 10-fold. To better understand these processes, including microbial degradation, future research should examine asparagus autotoxicity and the subsequent fate of *trans*-cinnamic acid exuded by asparagus plants in a field setting.

## 6. Interaction of Autotoxicity and Fusarium Infection

Plants have developed complex defense mechanisms to protect themselves, including constitutive and inducible defenses. Constitutive defenses include physical barriers, such as thick cell walls and waxy cuticles, as well as pre-existing compounds and enzymes that prevent infection by pathogens. Inducible defenses include cell wall modification, production of antibiotic compounds and reactive oxygen species, and the hypersensitive response (apoptosis). Phenylpropanoids, such as cinnamic acid, *p*-coumaric acid, caffeic acid, and ferulic acid, are reported to be inducible antibiotic compounds that serve as defense mechanisms in many plant species [164,165,166,167].

It has also been reported that asparagus produces these antibiotic compounds. Aqueous and organic solvent extracts of asparagus aerial parts and roots have been shown to suppress the growth of *F. oxysporum* [168,169]. Exudates from the surface of asparagus roots have also been shown to inhibit the spore germination and growth of *F. oxysporum* [170]. Among the potential asparagus autotoxic substances, *p*-coumaric acid, caffeic acid, and ferulic acid inhibited the spore germination and growth of *F. oxysporum* [171,172]. Ferulic acid also suppressed the expression of *FUM* genes, which are related to fumonisin biosynthesis, in *F. proliferatum* [173]. These findings suggest that *p*-coumaric acid, caffeic acid, and ferulic acid act as antibiotics by suppressing the spore germination, growth, and mycotoxin production of *Fusarium* species. However, it is unclear whether these compounds accumulated in the rhizosphere soil act as antibiotics in a defense mechanism of asparagus.

Both aqueous and organic solvent extracts of asparagus spears have been reported to stimulate fumonisin biosynthesis in *F. oxysporum* [174,175]. *Fusarium* pathogenicity increased significantly when sterilized asparagus crowns and roots were added to the soil during field cultivation [107]. Under greenhouse conditions, extracts from decaying asparagus roots increased *Fusarium* activity and exacerbated disease symptoms in asparagus plants [176]. These findings suggest that certain compounds in the asparagus increase the *Fusarium* pathogenicity.

Sterilizing asparagus crowns and roots did not affect the growth of these pathogenic *Fusarium* species. However, the crowns and roots were toxic to other fungi and soil microorganisms [107]. Based on this finding, it can be concluded that autotoxic substances may suppress beneficial soil microbes, but do not significantly impact these *Fusarium* species. This results in the dominance of pathogenic *Fusarium* species in asparagus rhizosphere soils, thereby exacerbating disease symptoms. However, the information on the effects of autotoxic substances on beneficial soil microbes and *Fusarium* is limited. Further investigation of this topic is necessary.

Both caffeic acid and ferulic acid have been reported to inhibit the growth of asparagus. This growth inhibition exacerbates the disease symptoms of *Fusarium* root rot in asparagus seedlings [171]. Cinnamic acid increased the production of cell wall-degrading enzymes, including cellulase, by *F. oxysporum* during pathogenesis [177,178]. Plant cell walls play an important role in defense mechanisms against pathogen infection [179,180]. Cinnamic acid also inhibited the induction of plant defense enzymes such as chitinase [177]. Plant chitinase, induced by a pathogenic infection, breaks down the glycosidic linkage in chitin. Chitin is a primary component of the cell walls of pathogenic fungi. Therefore, plant chitinase protects against fungal infection [181,182]. Inhibiting chitinase induction by cinnamic acid reduced the defense mechanism of plants, resulting in enhanced *Fusarium* pathogenicity [177]. These authors did not distinguish between *cis*- and *trans*-cinnamic acids. However, based on their research practices, it could be *trans*-cinnamic acid.

Although cinnamic, *p*-coumaric, caffeic, and ferulic acids have been reported to act as antibiotics, these compounds can also increase the pathogenicity of *Fusarium*. Cinnamic acid disrupts the defense mechanisms of host plants and stimulates the production of cell wall-degrading enzymes in pathogenic fungi. This makes host plants more susceptible to *Fusarium* infection. Thus, the presence of *Fusarium* and autotoxic substances creates a vicious cycle that exacerbates the “asparagus decline” and “asparagus replant problem”. Replanting young asparagus plants in an old asparagus field results in high mortality due to the presence of *Fusarium* and autotoxic substances existed in the old asparagus field. However, conflicting information exists regarding the effectiveness of these autotoxic substances in stimulating pathogenesis or protecting against *Fusarium* infection. Therefore, further research is needed to investigate the interaction of these autotoxic substances in the defense mechanism and pathogenesis of *Fusarium* under field conditions. Table 1 summarizes the substances that cause asparagus autotoxicity and their mechanisms of action.

## 7. Management of Autotoxicity and Fusarium Infection

Many researchers have investigated the effectiveness of activated carbon and biochar in reducing asparagus autotoxicity. Mixing activated carbon or biochar into the asparagus-cultivating soil or injecting flowable activated carbon into the soil increased the growth and productivity of asparagus compared to the control group [183,184,185,186,187]. Under hydroponic conditions, titanium dioxide (TiO_2_) with UV light irradiation reduced the autotoxicity of asparagus. This may be due to the photocatalytic decomposition of toxic substances [188]. However, these treatments only partially recovered the asparagus autotoxicity.

Various management strategies have also been employed to mitigate *Fusarium* infections and diseases. These strategies include inoculating nonpathogenic *Fusarium* species into soil [189], inoculating arbuscular mycorrhizae [190,191], incorporating asparagus root residues into the soil [192], mixing sodium chloride into the soil [193,194], and performing biofumigation [195,196,197,198]. These treatments have exhibited varying levels of efficiency, with some being labor-intensive [39]. Several fungicides, including benomyl, thiophanate-methyl, and fludioxonil, were insufficiently effective at overcoming diseases caused by *F. oxysporum* and *F. proliferatum* under growth chamber, greenhouse, and/or field conditions [189,190,199,200]. Herbicides are often applied to asparagus fields to control weeds. However, these herbicide treatments increase the risk of *Fusarium* infection in asparagus [39], Breeding *Fusarium*-resistant cultivars may be an efficient way to reduce the infection [201,202]. However, commercially viable, resistant cultivars have not yet been produced. Consequently, there is currently no single, effective control measure available to combat *Fusarium* infection. *Fusarium* infection and autotoxic substances may work together to suppress the growth and productivity of asparagus. One solution is to rotate asparagus crops with other crops [111,203,204].

As described in Section 2, autotoxicity generally acts as a form of self-regulation. Examples include adjusting population density to prevent intense intraspecific competition, suppressing germination during adverse environmental conditions, and promoting species migration to new habitats. Autotoxicity also increases species diversity by suppressing host plant species [78,87,88,104]. However, the ecological significance of asparagus autotoxicity is still unknown. This phenomenon should be investigated in wild asparagus populations in their natural habitats. Such research could provide valuable insight into addressing the “asparagus decline” and “asparagus replant problem”.

## 8. Conclusions

Based on the literature review, asparagus is autotoxic and autotoxic substances may be released into the soil through leaching, root exudation, and the decomposition process of asparagus residues. Several phenylpropanoids, including *trans*-cinnamic acid, *p*-coumaric acid, caffeic acid, and ferulic acid, have been identified as autotoxic substances by different research. Tryptophan, 3,4-methylenedioxycinnamic acid, and iso-agatharesinol were also identified as autotoxic substances in asparagus. Among them, *trans*-cinnamic acid was found in asparagus tissues, root exudates, rhizosphere soils, and growth medium. When exposed to UV light in sunlight, *trans*-Cinnamic acid undergoes photoisomerization, converting it into *cis*-cinnamic acid. This isomerization increases its growth inhibitory activity by over 10-fold. These substances may cause autotoxicity by disrupting phytohormone levels and cellular metabolism, and by impairing membrane function and inducing oxidative stress. They also weaken the defense mechanisms of asparagus against pathogen infection, and enhance *Fusarium* pathogenicity. The presence of these autotoxic substances and *Fusarium* infection creates a vicious cycle that worsens the “asparagus decline” and “asparagus replant problem”. To better understand asparagus autotoxicity, however, the concentrations of these compounds in asparagus root exudates, residues, and rhizosphere soils in fields should be determined. Additionally, the ecological significance of asparagus autotoxicity should be investigated in the future.

## Figures and Tables

**Table 1:** Asparagus autotoxic substances and their mechanisms of actions.

Autotoxic Substance	Mechanism of Action	Reference
*trans*-Cinnamic acid	Induction of oxidative stress. Interruption of auxin biosynthesis. Disruption of cell membranes and defense function. Enhance *Fusarium* pathogenicity.	[151,152,153,154,155,156,177,178]
*p*-Coumaric acid	Induction of oxidative stress. Disruption of cell membranes and photosynthesis.	[135,142]
Caffeic acid	Induction of oxidative stress. Interruption of gibberellic acid biosynthesis and MAPK signaling pathway. Enhance *Fusarium* pathogenicity.	[135,136,171]
Ferulic acid	Disruption of cell membranes and walls, and photosynthesis. Inhibition of arbuscular mycorrhizal colonization. Enhance *Fusarium* pathogenicity.	[124,125,126,171]
Tryptophan	Interruption of auxin biosynthesis.	[129,131]
3.4-Methylenedioxycinnamic acid	Inhibition of 4-coumarate-CoA ligase	[120,121,122]
Iso-agatharesinol	unknown	---

## Data Availability

No new data were created or analyzed in this study.
